# The effect of exposure to the COVID-19 pandemic on nutritional status and cognitive, motor, and behavioural development among children aged 20 months in rural Bangladesh: A repeated cross-section study between 2020 and 2022

**DOI:** 10.1371/journal.pone.0309836

**Published:** 2025-03-18

**Authors:** Jena Derakhshani Hamadani, Saiful Alam Bhuiyan, Mohammed Imrul Hasan, Sally Grantham-McGregor, S.M. Mulk Uddin Tipu, Diego Parra Alvarez, Shamima Shiraji, Laura Becerra Luna, Syed Nazmul Huda, Norbert Rudiger Schady, Alaka Holla

**Affiliations:** 1 Maternal and Child Health Division, International Centre for Diarrhoeal Disease Research, Bangladesh (icddr,b), Dhaka, Bangladesh; 2 The University of Melbourne, Parkville, Victoria, Australia; 3 The Walter and Eliza Hall Institute of Medical Research, Parkville, Victoria, Australia,; 4 Institute of Child Health, UCL Great Ormond Street, London, United Kingdom; 5 The World Bank, Washington, DC, United States of America; National Research Centre, EGYPT

## Abstract

**Background:**

Past studies have documented detrimental effects of the COVID-19 pandemic on the learning and mental health of preschool- and school-age children. Few studies have examined effects on younger children’s development, though this age group is extremely sensitive to economic and health shocks.

**Methods:**

We assessed the effects of exposure to the pandemic on the cognitive, language, and motor development; behaviour; and growth among toddlers in rural Bangladesh. We estimated average differences between two repeated cross-sectional surveys of children and mothers living in the same villages. The first survey included 20-month-old children in 2019 and 2020 (unexposed group). The second survey took place in a randomly-selected subset of the same villages in 2022 among 20-month-old children, who had experienced pandemic-related lockdowns from approximately mid-gestation through their first year (exposed group). Both surveys used similar inclusion criteria and the same developmental assessments (Bayley’s Scales of Infant and Toddler Development), behaviour observations, and field protocols.

**Results:**

The exposed group (N = 526) had lower cognitive [Effect size = -0.45 (95% CI = -0.63 to -0.27)] and motor [-0.55 (-0.73 to -0.37)] composite scores, compared to the unexposed group (N = 1344). They were also observed to be less responsive to the examiner [-0.29 (-0.48 to -0.11)], less happy [-0.37 (-0.55 to -0.19)], less vocal [-0.57 (-0.73 to -0.4)] and less cooperative [-0.42 (-0.6 to -0.24)]. The pandemic increased depression among mothers with a primary education or less but not among better educated mothers. Children of less educated mothers also showed larger differences across exposed and unexposed groups in development and behaviour than those with better educated mothers.

**Conclusion:**

The COVID-19 pandemic detrimentally affected cognitive and motor skills and behaviour of young children in rural Bangladesh. Disadvantaged young children’s development appears to be extremely vulnerable to shocks. Without intervention these deficits will likely lead to later problems in learning and mental health.

## Introduction

With its accompanying movement restrictions, economic slowdowns, and service closures, the COVID-19 pandemic altered the environments in which very young children develop [1]. The pandemic and lockdowns led to learning loss [2,3] and deteriorations in mental health [4] in school-aged children globally. Children between 3 and 6 years of age attending pre-primary education also suffered learning losses as well as declines in motor and socio-emotional skills [5,6]. However, there has been scarce information on the development of children under 2 years following the pandemic. While children in this age group do not attend school, their overall development can be affected as the pandemic may have exposed them to increases in parental stress and economic insecurity at home, as well as a decline in health services.

Knowledge of the pandemic’s effects on children who are under age 2 years is critical, as brain development in the first few years of life is particularly sensitive to risk factors in the environment [7,8]. Skill deficits that arise during this period tend to persist into adulthood and sometimes amplify over time [9–12]. Children who were under age 2 when exposed to the pandemic are approximately two years away from entering primary school. An estimate of the prevalence of any deterioration in development and behaviour among this age group can help plan remediation efforts for improving child development before affected cohorts progress to primary schooling and preview any changes in the school curriculum or health services that may be required if deficits are not addressed within the next two years. Such estimates may be even more important among low-income populations in low- and middle-income countries where there is limited formal social assistance and families are more vulnerable and cannot fully insulate themselves from aggregate shocks, such as recessions or natural disasters [13–15].

Measuring the impact of the pandemic on very young children, however, is a challenge. Unlike for preschool- and school-age children, where there are routine tests of children’s development or school achievement available for both pre-pandemic and post-pandemic cohorts, frequent developmental assessments are not so readily available for children under age 24 months. The task of assessing children’s cognitive, language, motor, and socio-emotional skills at a young age is not easy. Full developmental assessments are the most sensitive and testers must be highly skilled to obtain scores that are both accurate and reliable [16]. Samples must often be identified through house-to-house surveys to avoid the bias that can arise from compositional changes in the populations attending facilities, such as clinics and day care centers, after lockdowns have been lifted.

A few studies compared young children born and reared in the pandemic with pre-pandemic cohorts [17–22], but they generally used screening tests such as the Ages and Stages Questionnaires [23], which are based on the reports of mothers collected routinely by clinic workers [19] or teachers at day care centers [21]. Moreover, the pre-pandemic cohort sometimes had different enrolment criteria from those children tested during or after the pandemic [17,20], and most came from high income countries [19–22] or China [18]. A Canadian study [22] found a decrease in vocabulary but only in children from low socio-economic families. An earlier study in urban slums in Bangladesh found pandemic effects on weight-for-height but not height-for-age in children under age 5 years, but cognitive, motor, and language development and behaviour were not reported [24]. We were unable to find any other studies estimating the impact of the pandemic on directly observed socio-emotional behaviour.

The aims of the current study were to (i) estimate the impact of the COVID-19 pandemic on very young children’s growth, development, and behaviour in a low-resource country, using full developmental assessments conducted by skilled testers in the context of a household survey among a population-based sample of 20-month children in rural Bangladesh and (ii) examine maternal education and gender as possible effect-modifiers of any impact.

### Materials and methods

In Bangladesh the first cases of COVID-19 were reported in March 2020, and the first lockdown occurred from 26^th^ March to 30^th^ May 2020, while other lockdowns were imposed from 5^th^ April to 24^th^ May 2021 and from 22^nd^ June 2021 in seven districts surrounding Dhaka, followed by a country wide lockdown from 1^st^ July to 10^th^ August 2021.

The study used a repeated cross-section design that compared two groups of 20-month-old children. The first group, surveyed in 2018, 2019, and early 2020, was unexposed to the COVID-19 pandemic in their first 20 months of life. That is, during their first 20 months of life, they were developing without the lockdowns and associated economic recessions brought on by the pandemic. The second group, surveyed from a random sample of the same villages in March to May 2022, using the same sampling protocol for children, had been exposed to the pandemic beginning from the third to sixth month of gestation through to approximately 12 months of age. That is, they were initially developing in utero and after birth during lockdowns and any associated financial stress. The unexposed group had been assessed as part of a follow-up survey, 9 months after a randomised controlled trial of iron supplementation ended. The iron supplementation had shown no effect on children’s developmental levels, behaviour or growth, with benefits evident only on blood values (Hb, iron status) [25]. Both groups were assessed using identical tests and field protocols.

### Groups

#### The unexposed group.

The unexposed group were formed from the endline of the iron supplementation trial. Children were initially enrolled at age 8 months ± 14 days into the iron supplementation trial, after having been identified through lists of children and pregnant women provided by government health workers and through a house-to-house survey in all 112 villages in three *unions*, the smallest rural administrative and local government units (Bhulta, Gulakandail, and Rupganj), which were selected from the Rupganj Upazila (sub-district) in Narayanganj District in central Bangladesh. All eligible children aged eight months ± 14 days and their mothers who resided in any of the three unions were surveyed, but children with the following characteristics were excluded from the trial: severe anemia (a haemoglobin level of < 8.0 g per decilitre, n = 52, 1.4%), severe acute malnutrition (mid upper arm circumference < 11.5 cm, n = 24, 0.6%), current febrile illness (n = 31, 0.77%), a known inherited red-cell disorder or previous transfusion (n = 1, 0.02%), known developmental delay (n = 20, 0.49%), or household drinking water with iron level exceeding 1 mg per liter (n = 130, 3.2%). Further details of the trial are available [25].

The unexposed group received three months of iron supplementation. Nutritional status, development, and behaviour of the entire experimental group were assessed at age 11 months at the end of the trial and 9 months later when they were age 20 months ± 14 days. The 20-month assessments, which were used in the present study, were completed from the 8^th^ of July 2018 to the 19^th^ of Feb 2020, finishing just before the COVID-19 pandemic began in Bangladesh.

#### The exposed group.

This group was formed in 2022 during another round of data collection from a set of 50 villages randomly sampled from all 112 villages included in the iron supplementation trial. Children in the target age range (20 months ± 14 days) were identified from similar lists to those used in the unexposed group, in addition to house-to-house surveys. All eligible children who were 20 months ± 14 days between the 22^nd^ of March and the 31^st^ of May 2022 and their mothers were selected, and exclusion criteria were nearly identical to those applied in the unexposed group, except children with severe acute malnutrition or severe anemia could not be excluded. As we only observed the exposed group at age 20 months, it was not possible to know what their iron and nutritional status had been when the children were eight months, when exclusion criteria were applied to the unexposed group. We take this small difference in exclusion criteria (affecting 2 percent of the sample) into account in the analyses. We dropped one village where we did not find an eligible child. It is important to note that children in the unexposed sample who were part of the iron supplementation trial could not be part of the exposed sample as they would have been too old – that is, they were already 20 months ± 14 days before the pandemic.

#### Sample size.

In the unexposed group, there were a total of 2750 children aged 20 months ± 14 days, who were tested in the 112 villages that were part of the iron supplementation trial. In order to complete data collection within 3 months in 2022, we randomly selected a subset of 50 villages for the 2022 sample. We used the unexposed sample to estimate the intra-cluster correlations (ICC) for all Bayley composite correlations for all Bayley composite scores (measures described in more detail below), using the *loneway* command in Stata. To ensure adequate sample, we chose the upper-bounds of the ICC confidence intervals in our power calculations, which we conducted using the *power twomeans, cluster* command in Stata, which is recommended for two-sample t-tests that account for clustering. For 95 percent confidence levels, these calculations indicated that to achieve at least 80 percent power, we would need 500 exposed children in the 50 villages with an average number of 10 children in each village to detect differences of at least 0.2 standard deviations in all three Bayley composite scores (cognitive, language, and motor) between the exposed and unexposed groups. We chose a minimum detectable effect size of 0.2 standard deviations because anything lower would be considered a modest effect-size in the early childhood developmental literature. From the unexposed group, we selected all 1344 children who were tested at age 20 months ± 14 days in the 49 villages in which we conducted the data collection in 2022. In the exposed group, a total of 536 children were screened for enrolment, but 10 children were excluded after screening due to refusal (n = 5), sickness at the time of test (n = 3), migration (n = 1), and the presence of a birth defect (n = 1), leaving a total of 526 children enrolled in the exposed group.

#### Primary outcomes.

**Development:** Cognitive, language, and motor development were assessed with the Bayley Scales of Infant and Toddler Development, third edition [26], which are considered the gold standard of developmental assessments internationally [16]. This test has been previously adapted for Bangladesh to be culturally appropriate [27]. Taking approximately 60 minutes to administer, the test consists of 91 items to assess cognitive development, 49 items for receptive language, 48 items for expressive language, 66 items for fine motor skills, and 72 items for gross motor skills. Trained enumerators must assess based on observation whether or not a child can do the tasks described by the items, such as sorting pegs by color or throwing a ball. Within each domain of development, tasks the child can do are added up and converted to a scaled score based on the child’s exact age. For the analysis, we used the Bayley cognitive, language, and motor *composite scores*, which are scaled using a population of children in the United States as the standardization sample so that each mean composite score is equal to 100 with a standard deviation of 15. Since the test has not been standardized for Bangladeshi children, we also internally standardized the raw scores using the mean and standard deviation of the raw scores of the unexposed group. The Bayley motor composite scores comprise both fine and gross motor subscales, and the language composite includes both receptive and expressive language scores. Thus, there are 5 raw scores for every test.

**Behaviour:** Children’s behaviour during the Bayley test was rated on four nine-point scales of the Wolke’s Behaviour Ratings [28]: (i) response to the tester in the first ten minutes, (ii) general emotional tone, (iii) cooperation with the tester, and (iv) vocalisation throughout the test.

**Anthropometry:** Anthropometry was measured using standard methods. Z-scores for height-for-age, weight-for-age, and weight-for-height were calculated using the 2006 WHO Child Growth Standards [29].

**Testers:** Six testers with a Master’s degree in Psychology or Social Sciences who had conducted the Bayley tests with children during the iron supplementation trial were retrained for two weeks before implementing the 2022 round of data collection. They were unaware of the purpose of the study. Before the study began, each tester conducted between five to eleven tests in the presence of trainers. According to intraclass correlations (ICCs), inter-observer reliability before data collection was 100% for the Bayley scores and ranged from 0.71 to 0.97 for the behaviour ratings. During actual testing, 10% of measurements were observed by one of two trainers. Testers achieved 100% agreement with the trainers on developmental scores, while agreement for the behaviour ratings ranged from ICCs of 0.79 to 0.98.

#### Secondary outcomes.

**Family care indicators (FCI):** We assessed the quality of home stimulation and maternal depression, using the FCI [30], which has 20 items across four subscales of i) play activities, ii) play materials, iii) household books, magazines and newspapers, and iv) maternal depression that uses a short (6-item) version of the Center for Epidemiological Studies-Depression (CES-D) scale [31]. The FCI has been previously piloted and modified for rural Bangladeshi children [32].

**Food security:** We assessed food security within the household using the Household Food Insecurity Access Scale (HFIAS) [33], which consists of a set of 18 items that assess anxiety and uncertainty about household food supply, insufficient food quality, and insufficient food intake over the previous 30 days.

**Socio-economic status:** Information related to socio-economic background (such as housing conditions, parental education, and perceived financial security) was collected through a structured questionnaire that was used in the iron supplementation trial. To construct a housing index, we used the first principal component from indicators for the ownership of a house; the quality of materials used for the wall, roof, and floor of the house; and the presence of natural gas for cooking, a sanitary toilet, and piped drinking water. Similarly, we constructed an asset index using the first principal component from indicators for land ownership and the presence of a computer, fan, refrigerator, and electricity.

### Statistical analyses

We examined differences between the groups in both characteristics and outcomes with independent sample t-tests/Chi-square tests. We also estimated the effect of the pandemic on children’s development with multiple regression analysis, regressing the main outcomes (the Bayley subscales, behaviour ratings, and anthropometric variables) on an indicator for being in the exposed group, controlling for child age in days, child sex, the identity of the Bayley testers, and treatment status in the iron supplementation trial (for the unexposed group). We controlled for child age as children tend to pick up more skills with age, and we control for the identity of the Bayley testers to control for any consistent differences in how testers assess children. Though results of the iron supplementation trial suggested no significant improvement in children’s development, we controlled for unexposed children’s treatment status so that the average difference between the exposed and unexposed sample cannot be confounded with any effect, however small, generated by iron supplementation. In the unexposed group, children’s development scores and anthropometrics differed by geography (union) and by month of measurement (S1 Table in S1 Text). Though there was no consistent pattern across outcomes, we also included indicators for union and month of measurement in our regressions. We adjusted all corresponding p-values for multiple hypothesis testing with the Romano-Wolf correction, separately for the developmental composites, anthropometry, and behaviour [34].

In a separate analysis, we examined the nature and extent of heterogeneity in pandemic effects and estimated moderation effects for two characteristics that would not change in response to the pandemic – maternal education and child gender. To do this we added indicators for the moderators and an interaction between the potential moderator and the exposure indicator to our model.

We also examined the pandemic effect on secondary outcomes that could affect children’s development. These outcomes included food security, household financial security, and maternal depression.

As a robustness check, we included village fixed effects. This effectively restricted our comparisons between the exposed and unexposed groups to children within the same village, and the resulting differences were then averaged across villages.

We also checked whether differential application of exclusion criteria – specifically of not excluding severely malnourished or anaemic children in the exposed group - affected our results. The children in the unexposed group excluded due to poor nutritional status at age 8 months (2 percent of the original sample) may have had worse developmental outcomes than other children when observed at age 20 months. Therefore, their presence in the exposed group could have brought down the average of the exposed group compared to the unexposed group even in the absence of a pandemic effect. To test whether this underlies our results, we estimated Lee bounds, a method used to address differential attrition across treatment and control groups in a randomized controlled trial [35]. This method trims observations from the group with less attrition (in our case, the exposed group that has observations that would have been excluded in the unexposed group) so that the percentage of observations with observed outcomes is equal in both groups. Because we were worried that the exposed group may have had observations with worse outcomes solely due to the difference in exclusion criteria across the samples, we trimmed the two percent of exposed group with the worst outcomes and reran our main analysis.

We used Stata 17 for all analyses. All analytical code is available upon request.

### Ethical consideration

Proposals for both rounds of data collection were approved by the institutional review board of the International Centre for Diarrhoeal Disease Research, Bangladesh (icddr,b) (PR-16063 and PR-22027). Informed written consent was obtained from the guardians of the children before data collection.

## Results

Table 1 presents characteristics of the study groups. The exposed children were approximately three days older than the unexposed children. As child development tends to be positively correlated with age, this difference biases us against finding a disadvantage among the exposed group compared to the unexposed group. There were no significant differences across our two groups in socio-economic characteristics unlikely to be affected by the pandemic, such as the education of the sampled children’s parents, housing materials, and large assets, such as land and a refrigerator, and access to electricity.

**Table 1:** Characteristics of children and households in exposed and unexposed groups.

	Unexposed Group Mean (SD)/N(%)	Exposed Group Mean (SD)/n(%)	p Value (t-Test/Chi Square)
**Characteristics unlikely affected by the pandemic**			
**Age (Months)**	19.44 (0.54)	19.55 (0.54)	0.00
** Female**	654 (49%)	261 (50%)	0.86
** Male**	681 (51%)	265 (50%)	.
**Maternal Education, Years**			0.55
** 0—5**	384 (29%)	156 (30%)	.
** 6—9**	613 (46%)	228 (43%)	.
** 10—17**	337 (25%)	141 (27%)	.
**Paternal Education, Years**			0.81
** 0--5**	577 (43%)	210 (40%)	.
** 6--9**	429 (32%)	178 (34%)	.
** 10--17**	328 (25%)	131 (25%)	.
**Asset index**	0.04 (1.24)	0 (1.13)	0.93
**Housing index**	-0.08 (1.63)	0 (1.7)	0.93
**Characteristics possibly affected by the pandemic**			
**Cognitive composite score**	92.56 (8.31)	88.57 (8.66)	0.00
**Language composite score**	92.65 (9.69)	91.66 (8.98)	0.04
**Motor composite score**	96.12 (6.91)	91.91 (7.09)	0.00
**Approach**	5.52 (0.9)	5.17 (0.91)	0.00
**Cooperativeness**	5.52 (0.98)	5.26 (1.15)	0.00
**Vocalization**	4.27 (1.26)	3.67 (1.2)	0.00
**General emotional tone**	5.63 (0.98)	5.33 (1.17)	0.00
**Weight-for-Age Z-Score (WAZ)**	-0.79 (1.06)	-0.94 (1.2)	0.01
**Height-for-Age Z-Score (HAZ)**	-1.36 (0.98)	-1.32 (1.18)	0.41
**Weight-for-Height Z-Score (WHZ)**	-0.17 (1.04)	-0.41 (1.18)	0.00
**FCI: Play Materials Score**	4.14 (1.12)	3.82 (1.21)	0.00
**FCI: Play Activities Score**	5.35 (2.87)	6.01 (3.38)	0.00
**FCI: Reading Materials Score**	9.27 (6.1)	7.77 (5.72)	0.00
**Maternal depression score**	6.65 (8.03)	8.49 (8.78)	0.00
**Food security (%)**	78 (0.41)	57 (0.49)	0.00
Monthly family income *	25435.6 (19293.7)	22325.2 (20610.4)	0.00
Monthly family expenditure*	21685.3 (25289.8)	19309. 0 (13524.2)	0.04
**Deficit: always present (%)**	6 (0.24)	15 (0.36)	0.00

Data are mean (SD) or N (%). The housing index included the ownership of a house, the quality of materials used for the wall, roof, and floor of the house, and the presence of natural gas for cooking, sanitary toilet, and piped drinking water. The asset index included the ownership of land and the presence of electricity, computer, fan, and refrigerator. * income in takas 2022 value.

Both the unexposed and exposed children exhibit developmental scores below the average of 100, but it is important to recall that the Bayley composite scores have been normed using a sample of children from the United States. Children exposed to the pandemic, however, were significantly worse in all developmental, behavioural, and anthropometric measurements except for height-for-age z scores compared to the unexposed. Likewise, children in rural Bangladesh are far below global standards for anthropometric measures, particularly in height-for-age.

When we estimated the pandemic effect in multiple regression analyses, children in the exposed group had significantly lower cognitive, language, and motor scores, compared to the unexposed group (Table 2). The magnitudes of the pandemic effects were similar when using the Bayley composite scores standardized by the United States’ standardization population or the raw scores standardized by the distribution of scores in the unexposed group (S2 Table in S1 Text). The only difference was that the receptive and expressive language scores when measured separately were not statistically different between the exposed and unexposed groups, though the language composite scores were different.

**Table 2:** Pandemic effects on children’s cognitive, language, and motor development; nutritional status; and behaviour.

Variable	Pandemic Effect B (95% ci)	Standardized effect B, (95% ci)	p-value	Corrected p-value
**Cognitive composite score**	-3.75 (-5.26 to -2.25)	-0.45 (-0.63 to -0.27)	0.000	0.020
**Language composite score**	-1.74 (-3.33 to -0.15)	-0.18 (-0.34 to -0.02)	0.032	0.039
**Motor composite score**	-3.81 (-5.03 to -2.59)	-0.55 (-0.73 to -0.37)	0.000	0.020
**Weight-for-age z-score (WAZ)**	-0.02 (-0.22 to 0.17)	-0.02 (-0.2 to 0.16)	0.813	0.804
**Height-for-age z-score (HAZ)**	0.05 (-0.14 to 0.24)	0.05 (-0.14 to 0.24)	0.589	0.804
**Weight-for-height z-score (WHZ)**	-0.08 (-0.27 to 0.12)	-0.07 (-0.26 to 0.11)	0.447	0.745
**Approach**	-0.27 (-0.43 to -0.1)	-0.29 (-0.48 to -0.11)	0.002	0.039
**Cooperativeness**	-0.41 (-0.59 to -0.24)	-0.42 (-0.6 to -0.24)	0.000	0.020
**Vocalization**	-0.71 (-0.92 to -0.51)	-0.57 (-0.73 to -0.4)	0.000	0.020
**General emotional tone**	-0.37 (-0.54 to -0.19)	-0.37 (-0.55 to -0.19)	0.000	0.020

The pandemic effect B is the regression coefficient and 95% confidence limits of a regression of the outcome variable on an indicator for being in the exposed group and on controls for age of the child, sex of the child, maternal education, testers, month of measurement, location (union), and treatment status in an earlier iron supplementation trial. Standardized effect B size refers to results when the outcome variable is standardized using the unexposed distribution. There were 1344 children in the unexposed group and 526 children in the exposed group. P-values have been corrected for all outcomes with the Romano-Wolf procedure separately for composites, anthropometry, and behaviour.

Exposed children were also less responsive to the examiner, less happy, less vocal, and less cooperative during testing. Vocalisation showed the largest effect (-0.57SD). There were no statistically significant differences between the groups in any of the anthropometric measures.

Table 3 presents regression results for our secondary outcomes. Compared to the unexposed group, the exposed group was more food insecure and more likely to report a deficit between their incomes and their expenditures. However, the groups were not different on average in the frequency of play between caregivers and children, the availability of reading and play materials, and maternal depression.

**Table 3:** Pandemic effects on stimulation activities and materials, maternal depression, and household food and financial security.

	Pandemic Effect B (95% CI)	Standardized effect B 95% CI	P-Value	Corrected P-Value
**FCI: Play materials score**	-0.15 (-0.35 to 0.05)	-0.13 (-0.31 to 0.05)	0.15	0.67
**FCI: Play activities score**	0.22 (-0.34 to 0.79)	0.08 (-0.12 to 0.28)	0.44	0.84
**FCI: Reading materials score**	-1.26 (-2.35 to -0.18)	-0.21 (-0.39 to -0.03)	0.02	0.18
**Maternal depression score**	1.12 (-0.42 to 2.66)	0.14 (-0.05 to 0.33)	0.15	0.67
**Food security**	-0.31 (-0.37 to -0.24)	-0.75 (-0.9 to -0.59)	0.00	0.02
**Monthly family income (2022 Taka)**	-1401.6 (-4521.09 to 1717.88)	-0.07 (-0.23 to 0.09)	0.38	0.57
**Monthly family expenditure (2022 Taka)**	-1278.65 (-4138.5 to 1581.2)	-0.05 (-0.16 to 0.06)	0.38	0.57
**Deficit: Always or occasionally present**	0.19 (0.13 to 0.25)	0.53 (0.36 to 0.7)	0.00	0.02
**Deficit: Always present**	0.11 (0.07 to 0.16)	0.47 (0.29 to 0.66)	0.00	0.02

The pandemic effect B is the regression coefficient and 95% confidence limits of a regression of the outcome variable on an indicator for being in the exposed group and on controls for age of the child, sex of child, maternal education, month of measurement, location (union), and treatment status in an earlier iron supplementation trial. The FCI and maternal depression variables were also adjusted for age of the child and the identity of the interviewers. Standardized effect B refers to results when the outcome variable is standardized using the unexposed distribution. There were 1344 children in the unexposed group and 526 children in the exposed group. P-values have been corrected for all 9 outcomes with Romano-Wolf procedure. All taka amounts have been expressed in the equivalent of 2022 taka.

### Heterogeneity

**Fig 1:**
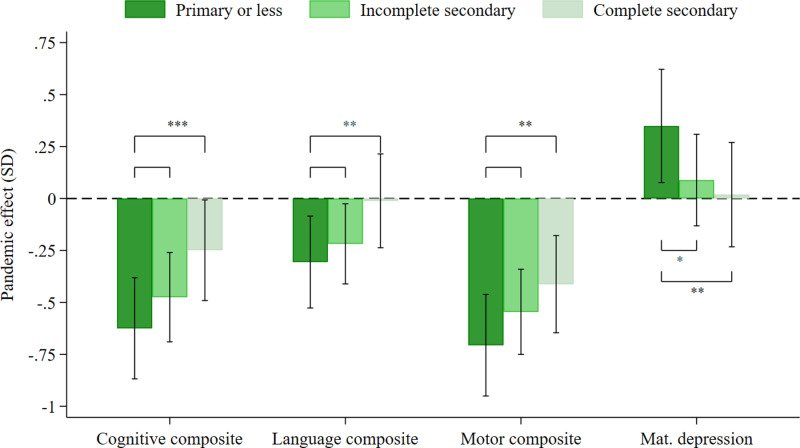
Pandemic effects on cognitive, language, and motor development, by maternal education. The graph plots pandemic effect sizes when using composite scores separately for three categories of children: (i) children with mothers whose highest level of completed education was primary or less (0-5 years of education), (ii) children whose mothers had an incomplete secondary education (6-9 years of education, and (iii) children whose mothers had completed secondary or higher education (10-17 years of education). Ranges shown with whiskers correspond to 95% confidence intervals. Significant differences in pandemic effect sizes between maternal education categories are indicated with *** (p < 0.01), ** (p < 0.05), and *  (p < 0.1). Data were assessed in 1,344 children in the unexposed group and 526 children in the exposed group.

We assessed whether maternal education moderated the effects of the pandemic on children’s development and maternal depression (Fig 1). The graph plots the effect size of the pandemic effect (reported in Table 2) separately for three groups: children with mothers whose highest level of completed education was primary or less (0-5 years), children whose mothers had an incomplete secondary education (6-9 years), and children whose mothers had completed secondary or higher education (10-17 years).

In all three maternal education groups, exposed children had significantly worse outcomes than unexposed children in the cognitive and motor scales. The pandemic effect was also significantly larger for children whose mothers had completed primary school or less compared to children whose mothers had completed secondary school or higher education. For language skills, significant pandemic effects were present only for children of mothers with primary school or fewer years of education and mothers with incomplete secondary school.

Though there was no significant pandemic effect on maternal depression in the full sample, Fig 1 shows that the least educated mothers experienced significant increases in depression, while pandemic effects were small and statistically insignificant among mothers with more than a primary education.

**Fig 2:**
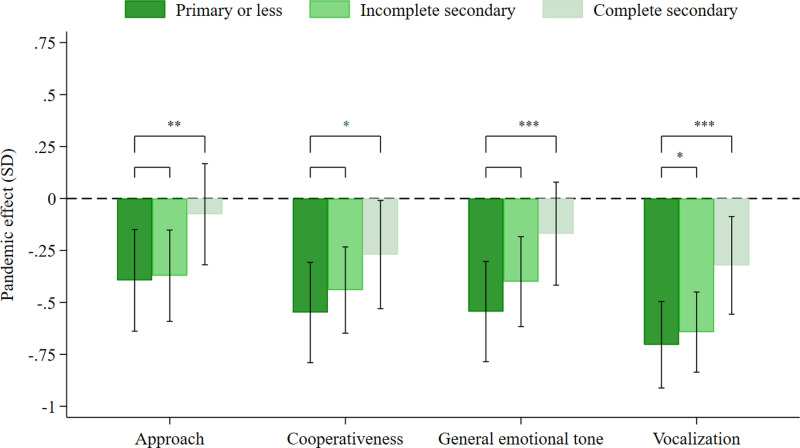
Pandemic effects on behavioral ratings, by maternal education. The graph plots pandemic effect sizes for behaviour ratings made by the testers, separately for three categories of children: (i) children with mothers whose highest level of completed education was primary or less (0-5 years of education), (ii) children whose mothers had an incomplete secondary education (6-9 years of education, and (iii) children whose mothers had completed secondary or higher education (10-17 years of education). Ranges shown with whiskers correspond to 95% confidence intervals. Significant differences in pandemic effect sizes between maternal education categories are indicated with *** (p < 0.01), ** (p < 0.05), and *  (p < 0.1). Data were assessed in 1,344 children in the unexposed group and 526 children in the exposed group.

We also assessed whether maternal education moderated the pandemic effect on the behaviour ratings assigned by the assessors. There was a significantly larger difference between the exposed and unexposed groups among children of less educated mothers compared to children with mothers who had completed secondary school or more in all 4 behaviours: approach, cooperativeness, general emotional tone, and vocalization (Fig 2).

Maternal education did not moderate the pandemic effect on food security or deficits between income and expenditure, nor did estimated impacts for male and female children statistically differ (results not reported here).

### Robustness

We checked the robustness of our results in two ways. First, we modified our main model to include village fixed effects (S3 Table in S1 Text). Estimated pandemic effects did not change significantly.

Second, we checked whether using different exclusion criteria across groups affected our results. To test whether the presence of exposed children who may have been severely anemic or malnourished at age 8 months underlies our results, we present upper Lee bounds, which represent pandemic effects after trimming the worst 2 percent of the observations from the exposed group (S4 Table in S1 Text). Even with this extreme assumption (that the children in the exposed group who would have been excluded in the unexposed group are the children in the exposed group with the worst outcomes), our results remain qualitatively unchanged.

## Discussion and conclusion

This study estimated pandemic effects on early cognitive, language, and motor skills and behaviour by comparing toddlers in rural Bangladesh who were unexposed to the pandemic with those exposed to the pandemic from mid-gestation through the first 12 months of age. These are one of the first estimates of the effect of the pandemic in such a young population in a lower-middle-income country that uses rigorous, full developmental assessments combined with direct observations of socio-emotional behaviour in a population-based sample. The findings are likely to be generalizable to the other rural regions in Bangladesh and possibly in other low- and middle-income countries, where households cannot fully insulate themselves from economic and health shocks [13,14].

The pandemic effects we estimated in cognitive and motor development were substantial, when benchmarked against findings from randomized controlled trials testing child development interventions targeting a similar age group. Specifically, the estimated effect size of the deficit in cognitive scores (-0.46 standard deviations (SDs)) was similar to the average estimated benefit in a meta-analysis of 32 parenting programs in low- and middle-income countries (average effect 0.41 SDs), while the pandemic effect on motor development (0.55 SD) is more than twice the average estimated effect size (0.26 SD, n = 29 studies) [36]. Motor deficits following the pandemic have also been found among preschool-age populations in other countries [5,6]. Evidence suggests these developmental deficits in development may lead to poorer learning and development at later ages [9].

Despite differences in reported food insecurity, children in the exposed group were not significantly more stunted or wasted than in the unexposed group. This finding is consistent with findings from the latest Joint Child Malnutrition Estimates of UNICEF, WHO, and the World Bank, which did not show increases in prevalence of stunting and wasting over the pandemic period [37].

All ratings of the children’s behaviours were also affected. The exposed children were more fearful when meeting the tester, less cooperative with the test procedure, and less happy throughout the test session. They also vocalised less. Overall, this suggests they were more inhibited and anxious, another finding consistent with pandemic effects measured on older children in preschools [5,6]. These early changes in mood and behaviour are concerning, as these behaviours may lead to later mental health problems [38].

We found no average effect on maternal depression, though many other studies have documented increases in both depression [39,40] and anxiety [40] among pregnant and post-partum women during the pandemic.

### Heterogeneity

The estimated deficits in cognition and motor development following the pandemic conceal a more important finding; the pandemic appeared to exacerbate inequality. The pandemic increased depression only among mothers with primary education or less, and their children were the only ones to show a deterioration in language skills due to the pandemic. Furthermore, they had sizeable cognitive (0.62 SD) and motor (0.71 SD) declines, which were larger than the declines experienced by children of better educated mothers (S5 Table in S1 Text).

The pandemic effects on children’s social-emotional behaviour were similarly moderated by maternal education, with children of mothers with low levels of education appearing to be more affected.

While higher maternal education partially protected children from developmental deficits, children of mothers who had completed secondary or higher education still experienced significant setbacks in cognitive (pandemic effect 0.25 SD) and motor (pandemic effect 0.41 SD) domains but not in language.

A possible explanation for the heterogeneity is that families with low educational attainment levels may have experienced different degrees of economic vulnerability during the pandemic. Unfortunately, we had no measures during the pandemic. In 2022, we found no differences across education groups in the pandemic’s effects on reported food and financial security.

Overall this pattern of findings is consistent with a recent review of pandemic effects on school-aged children, which found differential effects of the COVID-19 pandemic on learning loss and mental health by socio-economic status, with children from the poorest families suffering the greatest losses [41]. The cause of the pandemic effects found at school-age was generally attributed to loss of schooling, but here we show that even in infancy, when children are usually at home, effects on development appear to occur.

### Study strengths and limitations

A main strength of the present study was the similarity of the exposed and unexposed groups. Children in both groups came from the same villages and were restricted to a single age group (20 months), and their parents had similar levels of education, housing quality, and assets. The samples were identified by household surveys to address compositional changes that may be present in center-based samples. The unexposed group was tested two to three years before the exposed group. Therefore, the developmental differences we estimated between the groups are most likely due to the pandemic and its associated lockdowns.

The groups were also compared with well-established measurement tools using the same field protocols across surveys. The Bayley Scales of Infant and Toddler Development (3^rd^ edition) that we used are considered the “gold standard” for assessing child development. We used well trained testers who directly observed children rather than relying on reports by parents, who may not be reliable. We supplemented this tool with other well-established tools to measure children’s behaviour (Wolke’s Behaviour Ratings) and the home environment and maternal depression (Family Care Indicators).

The main limitation of the study is the number of data collection rounds. We only had one year of data for the unexposed and exposed groups. Having more rounds would have helped to demonstrate whether the pandemic represented a clear break in trend of average development scores at this age. Having more rounds of data collection may also have illuminated the mechanisms behind the substantial declines in development and behaviour. It would have been helpful, for example, to have had measures of maternal and environmental conditions during the pandemic, rather than just a snapshot in 2022.

### Implications

Developmental and behavioural deficits were present 7 to 9 months after the last lockdown, indicating that the deficits may not be transient. Past evidence suggests that in the absence of interventions, we might expect these early deficits to persist or even compound over time [9].

The findings demonstrate how economic and health shocks can have very different effects depending on the initial status of the exposed population, with the more disadvantaged being much more vulnerable than others. As future shocks are likely to occur more frequently with increasing climate change and conflict, assistance to high risk families with very young children should be prioritised. For example, parenting programs for families of the under three population [36,42], followed by preschool attendance [43], may help to reduce the effects these shocks have on children’s development.

## Supporting information

S1 Text Supplementary materials contain S1 Table, S2 Table, S3 Table, S4, Table, and S5 Table.
(DOCX)

S1 Data Data request form used by current research team to access 2018-2020 data. (DOCX)
