# Supplementary material for: The effect of exposure to the COVID-19 pandemic on nutritional status and cognitive, motor, and behavioural development among children aged 20 months in rural Bangladesh: A repeated cross-section study between 2020 and 2022
Source: PLoS One. 2025 Mar 18;20(3):e0309836. doi: 10.1371/journal.pone.0309836 (PMC11918444; doi:10.1371/journal.pone.0309836)
Supplement: S1 Text — (DOCX) [file pone.0309836.s001.docx]

# Supplemental materials

**S1 Table: Scores differed by region and by month of measurement in unexposed group**

|  | Cognitive | Language | Motor | WAZ | HAZ | WHZ | App | Coop | Vocal | Gen. Em |
| --- | --- | --- | --- | --- | --- | --- | --- | --- | --- | --- |
|  |  |  |  |  |  |  |  |  |  |  |
| Unions |  |  |  |  |  |  |  |  |  |  |
| Golakandail | 0.018 | -0.020 | -0.013 | -0.20** | -0.12 | -0.207** | -0.177** | -0.015 | 0.093 | -0.011 |
| Rupganj | 0.087 | 0.166** | 0.17** | 0.022 | -0.032 | 0.052 | -0.033 | 0.095 | 0.19*** | 0.18** |
|  |  |  |  |  |  |  |  |  |  |  |
| Months |  |  |  |  |  |  |  |  |  |  |
| February | -0.065 | 0.074 | 0.215* | -0.175 | -0.124 | -0.122 | -0.141 | -0.031 | -0.102 | 0.043 |
| March | 0.221 | 0.317** | 0.43*** | 0.018 | 0.066 | 0.010 | 0.134 | 0.147 | 0.220 | 0.194 |
| April | -0.081 | 0.005 | 0.088 | -0.163 | 0.217* | -0.32*** | -0.131 | 0.007 | -0.263** | -0.047 |
| May | 0.041 | -0.045 | 0.104 | -0.49*** | -0.034 | -0.60*** | -0.026 | 0.027 | -0.222** | -0.027 |
| June | -0.126 | -0.008 | 0.022 | -0.231 | 0.046 | -0.313** | -0.089 | 0.073 | -0.161 | -0.023 |
| July | 0.021 | -0.156 | 0.007 | -0.28** | -0.012 | -0.34*** | -0.31*** | -0.079 | -0.261** | -0.087 |
| August | -0.125 | -0.068 | 0.088 | -0.29** | -0.076 | -0.311** | 0.004 | 0.25** | 0.014 | 0.202 |
| September | -0.139 | -0.112 | -0.046 | -0.26** | 0.045 | -0.35*** | 0.261** | -0.034 | -0.199* | 0.118 |
| October | -0.256** | -0.220* | -0.165 | -0.155 | -0.041 | -0.145 | 0.039 | -0.116 | -0.40*** | -0.106 |
| November | -0.004 | 0.045 | 0.218* | -0.35*** | -0.23** | -0.29*** | 0.181 | 0.041 | -0.093 | 0.175 |
| December | 0.110 | 0.151 | 0.195* | -0.000 | -0.019 | 0.037 | 0.236** | 0.070 | -0.057 | 0.177 |
| Table shows the regression coefficient of a regression of the column variable on indicators for union and month of measurement, controlling for age of the child, sex of the child, identity of Bayley testers, and treatment status in an earlier iron supplementation trial. Data were assessed in 1344 children in the unexposed sample. *** P<0.01, ** P<0.05, * P<0.1  WAZ: Weight-For-Age z score, HAZ: Height-For-Age z score, WHZ: Weight-for-Height z score  App: Approach, Coop: cooperativeness, Vocal: vocalization, Gen Em: general emotional tone | | | | | | | | | | |
|  |  |  |  |  |  |  |  |  |  |  |
|  |  |  |  |  |  |  |  |  |  |  |

**S2 Table: Pandemic effects on raw scores, internally standardized**

|  | Pandemic Effect B (95% CI) | Standardized effect B (95% CI) | P-Value | Corrected P-Value |
| --- | --- | --- | --- | --- |
| Cognitive | -1.42 (-2.06 to -0.78) | -0.4 (-0.58 to -0.22) | 0.000 | 0.020 |
| Receptive Language | -0.32 (-0.77 to 0.12) | -0.12 (-0.29 to 0.04) | 0.151 | 0.255 |
| Expressive Language | -0.26 (-0.83 to 0.31) | -0.07 (-0.24 to 0.09) | 0.371 | 0.392 |
| Fine Motor | -0.52 (-0.88 to -0.17) | -0.27 (-0.44 to -0.09) | 0.004 | 0.020 |
| Gross Motor | -1.06 (-1.39 to -0.73) | -0.52 (-0.69 to -0.36) | 0.000 | 0.020 |
| Raw scores of the Bayley scales of infant and toddler development were compared and effect sizes were estimated using the mean and standard deviation of the raw scores in the unexposed sample. The pandemic effect B is the regression coefficient of a regression of the outcome variable on an indicator for being in the exposed group and controls for age of the child, sex of the child, maternal education, Bayley testers, month of measurement, location (union), and treatment status in an earlier iron supplementation trial. Standardized effect B refers to results when the outcome variable is standardized using the unexposed distribution. There were 1344 children in the unexposed sample and 526 children in exposed sample. P values have been corrected for all 5 outcomes with the Romano-Wolf procedure. | | | | |

**S3 Table: Pandemic effects on children’s development, village fixed effects**

|  | Pandemic Effect B (95% CI) | Standardized Effect B (95% CI) | P-Value | Corrected P-Value |
| --- | --- | --- | --- | --- |
| Cognitive composite score | -3.71 (-5.25 to -2.16) | -0.45 (-0.63 to -0.26) | 0.000 | 0.020 |
| Language composite score | -1.79 (-3.44 to -0.15) | -0.19 (-0.36 to -0.02) | 0.033 | 0.059 |
| Motor composite score | -3.58 (-4.83 to -2.32) | -0.52 (-0.7 to -0.34) | 0.000 | 0.020 |
| Weight-for-age z-score (WAZ) | 0.07 (-0.13 to 0.27) | 0.06 (-0.13 to 0.25) | 0.519 | 0.843 |
| Height-for-age z-score (HAZ | 0.11 (-0.08 to 0.31) | 0.11 (-0.08 to 0.31) | 0.256 | 0.510 |
| Weight-for-height z-score (WHZ) | 0.01 (-0.19 to 0.22) | 0.01 (-0.19 to 0.21) | 0.917 | 0.961 |
| Approach | -0.24 (-0.41 to -0.06) | -0.26 (-0.46 to -0.07) | 0.008 | 0.098 |
| Cooperativeness | -0.38 (-0.56 to -0.2) | -0.38 (-0.57 to -0.2) | 0.000 | 0.020 |
| Vocalization | -0.68 (-0.9 to -0.47) | -0.54 (-0.71 to -0.37) | 0.000 | 0.020 |
| General Emotional Tone | -0.34 (-0.52 to -0.15) | -0.34 (-0.53 to -0.16) | 0.000 | 0.020 |
| The pandemic effect B is the regression coefficient of a regression of the outcome variable on an indicator for being in the exposed group and indicators for age of the child, sex of the child, maternal education, identify of Bayley testers, month of measurement, location (Union), and treatment status in an earlier iron supplementation trial. The empirical specification also has village fixed effects. Standardized effect B refers to results when the outcome variable is standardized using the unexposed distribution. There were 1344 children in the unexposed sample and 526 children in exposed sample. P values have been corrected for all outcomes with the Romano-Wolf procedure separately for composites, anthropometry, and behaviour. | | | | |
|  |  |  |  |  |
|  |  |  |  |  |
|  |  |  |  |  |
|  |  |  |  |  |

**S4 Table: Pandemic effects on children’s development, adjusting for differential exclusion across cohorts**

|  | Pandemic Effect B (95% CI) | Standardized Effect B (95% CI) |
| --- | --- | --- |
| Cognitive composite score | -3.44 (-4.34 to -2.55) | -0.41 (-0.52 to -0.31) |
| Language composite score | -0.53 (-1.47 to 0.41) | -0.03 (-0.13 to 0.07) |
| Motor composite score | -3.79 (-4.49 to -3.08) | -0.47 (-0.57 to -0.37) |
| Weight-for-age z-score (WAZ) | -0.1 (-0.22 to 0.02) | . |
| Height-for-age z-score (HAZ) | 0.11 (0 to 0.23) | . |
| Weight-for-height z-score (WHZ) | -0.17 (-0.29 to -0.06) | . |
| Approach | -0.31 (-0.4 to -0.21) | -0.34 (-0.45 to -0.24) |
| Cooperativeness | -0.21 (-0.33 to -0.1) | -0.16 (-0.28 to -0.05) |
| Vocalization | -0.57 (-0.69 to -0.44) | -0.21 (-0.3 to -0.11) |
| General Emotional Tone | -0.26 (-0.37 to -0.14) | -0.17 (-0.29 to -0.06) |
| Lee bounds for non-random sample selection were used to adjust estimated pandemic effects for differences in exclusion criteria across the exposed and unexposed groups. In particular, the upper limit Lee bounds were used to estimate effects when the worst 2 percent of the exposed sample were trimmed from the sample (corresponding to the two percent excluded for iron deficiency and severe malnutrition at age 8 months in the unexposed group who could not be excluded in the exposed group). After this adjustment, the pandemic effect B is the regression coefficient of a regression of the outcome variable on an indicator for being in the exposed group. Standardized effect B refers to results when the outcome variable is standardized using the unexposed distribution. There were 1344 children in the unexposed group and 526 children in exposed group. | | |
|  | | |

**S5 Table: Pandemic effects on children’s development and behavior, by maternal education**

|  | **Variables** | **Maternal depression** | **Composites** | | | **Behavioural ratings** | | | |
| --- | --- | --- | --- | --- | --- | --- | --- | --- | --- |
|  |  |  | **Cognitive** | **Language** | **Motor** | **Approach** | **Cooperativeness** | **Vocalization** | **General emotional tone** |
| **Estimates** |  |  |  |  |  |  |  |  |  |
| B0 | Lockdown | 0.349** | -0.625*** | -0.306*** | -0.706*** | -0.394*** | -0.549*** | -0.704*** | -0.544*** |
|  |  | (0.139) | (0.124) | (0.113) | (0.124) | (0.125) | (0.123) | (0.106) | (0.123) |
| B1 | Ed: Incomplete Secondary | -0.131* | 0.099 | 0.208*** | 0.083 | 0.107* | 0.050 | 0.100 | 0.049 |
|  |  | (0.070) | (0.065) | (0.062) | (0.063) | (0.064) | (0.068) | (0.062) | (0.068) |
| B2 | Lockdown * Ed: Incomplete Secondary | -0.261* | 0.150 | 0.088 | 0.161 | 0.022 | 0.108 | 0.061 | 0.144 |
|  |  | (0.137) | (0.126) | (0.111) | (0.121) | (0.123) | (0.125) | (0.108) | (0.128) |
| B3 | Ed: Complete Secondary | -0.188** | 0.138* | 0.352*** | 0.151* | 0.152** | 0.095 | 0.157** | 0.085 |
|  |  | (0.076) | (0.075) | (0.074) | (0.077) | (0.074) | (0.079) | (0.074) | (0.078) |
| B4 | Lockdown * Ed: Complete Secondary | -0.331** | 0.376*** | 0.295** | 0.294** | 0.318** | 0.279* | 0.382*** | 0.375*** |
|  |  | (0.148) | (0.139) | (0.129) | (0.136) | (0.134) | (0.147) | (0.129) | (0.142) |
| B5 | Age (Days) | 0.001 | -0.004* | -0.001 | -0.002 | 0.004* | 0.004 | 0.011*** | 0.005** |
|  |  | (0.002) | (0.002) | (0.002) | (0.002) | (0.002) | (0.002) | (0.002) | (0.002) |
| B6 | Sex (Female = 1) | 0.028 | 0.120** | 0.250*** | 0.209*** | 0.157*** | 0.182*** | 0.212*** | 0.171*** |
|  |  | (0.048) | (0.047) | (0.044) | (0.045) | (0.046) | (0.048) | (0.045) | (0.048) |
| **Joint Effects** | |  |  |  |  |  |  |  |  |
| B0 | Ed: Primary or less | 0.349** | -0.625*** | -0.306*** | -0.706*** | -0.394*** | -0.549*** | -0.704*** | -0.544*** |
|  | P-value | 0.012 | 0.000 | 0.007 | 0.000 | 0.002 | 0.000 | 0.000 | 0.000 |
| B0 + B2 | Ed: Incomplete Secondary | 0.089 | -0.475*** | -0.218** | -0.546*** | -0.372*** | -0.441*** | -0.643*** | -0.4*** |
|  | P-value | 0.431 | 0.000 | 0.026 | 0.000 | 0.001 | 0.000 | 0.000 | 0.000 |
| B0 + B4 | Ed: Complete Secondary | 0.018 | -0.249** | -0.011 | -0.412*** | -0.076 | -0.27** | -0.322*** | -0.169 |
|  | P-value | 0.886 | 0.044 | 0.922 | 0.001 | 0.542 | 0.042 | 0.007 | 0.181 |
| **Fixed Effects** | |  |  |  |  |  |  |  |  |
|  | Treatment | Yes | Yes | Yes | Yes | Yes | Yes | Yes | Yes |
|  | Union | Yes | Yes | Yes | Yes | Yes | Yes | Yes | Yes |
|  | Month | Yes | Yes | Yes | Yes | Yes | Yes | Yes | Yes |
|  | Tester | Yes | Yes | Yes | Yes | Yes | Yes | Yes | Yes |
|  | Constant | -0.434 | 2.217* | 0.354 | 0.945 | -2.340* | -2.145 | -6.435*** | -2.732** |
|  |  | (1.306) | (1.226) | (1.161) | (1.270) | (1.312) | (1.346) | (1.159) | (1.337) |
|  | Observations | 1,815 | 1,859 | 1,859 | 1,859 | 1,858 | 1,858 | 1,858 | 1,858 |
|  | R-squared | 0.044 | 0.085 | 0.073 | 0.134 | 0.074 | 0.075 | 0.111 | 0.091 |
| Robust standard errors in parentheses: *** p<0.01, ** p<0.05, * p<0.1. Joint effects are estimated using the delta method, and the associated p-value is reported. | | | | | | | | | |
