# Supplementary material for: The effect of exposure to the COVID-19 pandemic on nutritional status and cognitive, motor, and behavioural development among children aged 20 months in rural Bangladesh: A repeated cross-section study between 2020 and 2022
Source: PLoS One. 2025 Mar 18;20(3):e0309836. doi: 10.1371/journal.pone.0309836 (PMC11918444; doi:10.1371/journal.pone.0309836)
Supplement: S1 Data — (DOCX) [file pone.0309836.s002.docx]

| Committee | A/Prof Sant-Rayn Pasricha, Walter and Eliza Hall Institute of Medical Research, Australia  Prof Beverley-Ann Biggs, Peter Doherty Institute for Infection and Immunity, Australia  Dr Jena Hamadani, International Centre for Diarrhoeal Disease Research, Bangladesh |
| --- | --- |
| Statistician | Ms Sabine Braat, University of Melbourne, Australia |

**How to request data from the BRISC study database?**

1. Define your research objective
2. Describe the planned (statistical) analyses, specifically indicate if the analysis will be per arm
3. List the variables of interest to extract from the main BRISC trial database
4. Specify your project timelines (e.g. number of weeks, months) or deadlines
5. List who besides yourself will need access to the requested variables
6. List who are the proposed authors on the manuscript
7. Provide any other information that you think may be relevant to support the request
8. Specify the dataset format (options: spreadsheet (xls), text (csv), Stata (dta))
9. Specify preferred sharing (options: WEHI or Doherty shared drive or temporary Dropbox folder)
10. If analyses is to be per arm, attach a statistical analysis plan (more detailed than above).

Email the above to Santa ([pasricha.s@wehi.edu.au](mailto:pasricha.s@wehi.edu.au)), Bev ([babiggs@unimelb.edu.au](mailto:babiggs@unimelb.edu.au)), Jena ([jena@icddrb.org](mailto:jena@icddrb.org)) with Cc Sabine ([s.braat@unimelb.edu.au](mailto:s.braat@unimelb.edu.au)).

After review, you will receive:

1. Feedback on your proposal and approval (yes/no) – from Santa, Bev, Jena.
2. Guidance on the proposed authorship – from Santa, Bev, Jena.
3. Review of the statistical analysis plan – from Sabine.
4. The requested variables in one or more (password protected) datasets in the format specified via the preferred file sharing platform – from Sabine.

When your project is finished, please provide Sabine with a copy of your final data (e.g. microbiome, EEG) for a centralised backup on our servers.

**General information on study database**

| Raw  Database* | Master trial database (consisting of collected data) stored at servers in:   - International Centre for Diarrhoeal Disease Research (Jena Hamadani) - Peter Doherty Institute for Infection and Immunity (Beverley-Ann Biggs) - Walter and Eliza Hall Institute of Medical Research (Sant-Rayn Pasricha) |
| --- | --- |
| Analysis  Database* | Analysis trial database (consisting of collected and derived data) stored at servers in:   - Peter Doherty Institute for Infection and Immunity (Beverley-Ann Biggs) - Walter and Eliza Hall Institute of Medical Research (Sant-Rayn Pasricha) |
| * Excludes e.g. microbiome, EEG, CyTOF, and other outcomes obtained not via study questionnaires | |

**Material available upon request to support request:**

1. Study protocol
2. Study questionnaires
3. Data dictionary

**Request form to be completed**

| **Research objective:** |
| --- |
| **Planned statistical analyses:**  **Per arm: Yes/No** |
| **Requested variables of BRISC trial database:** |
| **Project timelines/deadlines:** |
| **Who needs access:** |
| **Proposed authors manuscript:** |
| **Dataset format (options: spreadsheet (xls), text (csv), Stata (dta)):** |
| **Preferred file sharing platform (options: WEHI/Doherty shared drive or temporary Dropbox folder):** |
| **Other relevant information:** |
| Attach:   - If analyses is to be per arm, attach a statistical analysis plan (more detailed than above). |
